# Development of Poly(lactic acid)/Poly(3-hydroxybutyrate-co-3-hydroxyvalerate) Biocomposite Films: Influence of Cellulose Microfiber Source on Structural and Functional Properties

**DOI:** 10.3390/polym18111350

**Published:** 2026-05-29

**Authors:** Luis Jaime Pérez-Córdoba, Diana Carmona-Cantillo, Cristian Polo-Zamora, Edwin Fuentes-Ordóñez, Rodrigo Ortega-Toro

**Affiliations:** 1 Food Packaging and Shelf-Life Research Group (FP&SL), Food Engineering Department, Universidad de Cartagena, Avenida del Consulado Calle 30 No. 48–152, Cartagena de Indias 130015, Colombia; ljperezcordoba@gmail.com (L.J.P.-C.); dcarmonac1@unicartagena.edu.co (D.C.-C.); 2 Grupo de Investigación en Transformación Aplicada a Matrices Industriales y Agroindustriales—ITMIA, University of Cartagena, Cartagena de Indias 130001, Colombia; cpoloz@unicartagena.edu.co (C.P.-Z.); efuenteso@unicartagena.edu.co (E.F.-O.); 3 Research Group of Complex Fluid Engineering and Food Rheology (IFCRA), Food Engineering Department, Universidad de Cartagena, Avenida del Consulado Calle 30 No. 48–152, Cartagena de Indias 130015, Colombia

**Keywords:** biocomposite films, cassava, yam, potato, hulls, cellulose microfiber, melt extrusion

## Abstract

The incorporation of cellulosic-based fillers as reinforcements into biocomposites represents a promising strategy to enhance the performance of sustainable packaging materials. In this study, poly(lactic acid)/poly(3-hydroxybutyrate-co-3-hydroxyvalerate) (PLA/PHBV) films reinforced with 1 and 3 wt% of cellulose microfibers (CM) derived from yam, potato, and cassava hulls were developed through melt extrusion followed by compression molding. The physicochemical, mechanical, optical, microstructural, thermal, and molecular properties of the films were evaluated. Results showed that both the CM source and concentration significantly influenced the biocomposites performance. Cassava-derived CM at 3 wt% provided the best barrier properties, while increasing CM content, regardless of the source, generally reduced solubility, increased moisture content, enhanced stiffness, and decreased elongation at break, although excessive loading negatively affected structural homogeneity. CM incorporation also reduced film gloss and transparency, particularly in yam-based composites. Thermal analysis indicated a multi-step degradation process with only minor variations in thermal stability, and no major chemical modifications of the biocomposites were detected. Overall, cassava-derived CM produced the most balanced performance, highlighting the importance of filler source and loading in tailoring PLA/PHBV biocomposite functional properties.

## 1. Introduction

The growing environmental concerns associated with petroleum-based plastics have accelerated the development of bio-based polymeric materials as sustainable alternatives. Among these, poly(lactic acid) (PLA) and poly(3-hydroxybutyrate-co-3-hydroxyvalerate) (PHBV) have gained significant attention due to their biodegradability, biocompatibility, and potential to be processed using conventional thermoplastic techniques [1]. Despite their environmental advantages, both polymers exhibit intrinsic limitations such as brittleness, low impact resistance, limited thermal stability, and restricted barrier properties, which hinder their broader industrial application [2].

Blending PLA with PHBV has been widely proposed as a strategy to overcome these shortcomings by exploiting their complementary properties, since PLA provides strength, while PHBV enhances the crystallization behavior, toughness, and biodegradability in PLA-based systems. Nevertheless, PLA/PHBV blends often suffer from partial immiscibility and weak interfacial interactions, which limit the efficiency of stress transfer and ultimately compromise the mechanical performance of the resulting materials [3]. Consequently, reinforcing that blend with bio-based fillers has become a key research direction to tailor their structural and functional properties. For example, microfibers can act as a bridge or nucleating agent to stabilize the interface between these two polymers [4].

Cellulose fibers have emerged as promising reinforcing fillers due to their abundance in nature, renewability, low density, high aspect ratio, and their exceptional physical and mechanical qualities, which may be attributed to the presence of many hydroxy groups and an effective hydrogen bonding network [5]. Additionally, cellulose offers a unique combination of crystalline and hierarchical structure that can significantly contribute to reducing the overall environmental footprint of composite materials while enhancing their functional properties [6]. Jacob et al. [5] reported that cellulose incorporation into PLA-based systems can improve stiffness, crystallinity, and barrier properties, while maintaining biodegradability. Moreover, several studies have explored the incorporation of natural fibers into biopolymer matrices to develop biocomposites with improved mechanical and functional performance [7]. Marmol et al. [8] reported enhanced mechanical, water and UV-vis light barrier properties, and increased thermal stability in polyhydroxyalkanoate (PHA)-based films reinforced with CM obtained by a straightforward water dispersion of kraft paper. Similarly, Panaitescu et al. [9] observed significant improvements in the thermal and mechanical performance of PHB biocomposites reinforced with cellulose fibers derived from wood waste [7].

The incorporation of CM within the PLA/PHBV matrix has shown potential to improve mechanical strength, thermal resistance, and barrier performance. Nevertheless, the effectiveness of reinforcement strongly depends on factors such as microfiber dispersion, interfacial adhesion, manufacturing process, and especially, the CM source and concentration [7]. At low concentrations, CM can act as effective stress-transfer agents, whereas at higher loadings, microfiber agglomeration and poor interfacial compatibility may negatively affect the composite properties [10]. Bazan et al. [3] have demonstrated that increasing cellulose content in PLA/PHBV composites improves stiffness but reduces ductility and energy absorption capacity, highlighting a critical trade-off between rigidity and toughness.

In recent years, growing attention has been directed toward the valorization of agricultural residues as sustainable feedstock for cellulose fiber production. For instance, yam (Y), potato (P), and cassava (C) hulls are generated in substantial quantities as by-products during peeling and processing operations in the agri-food industry, representing between 10 and 30% of the total tuber mass, depending on the species and processing conditions. However, those hulls are often underutilized or discarded as waste [11]. Some studies have highlighted the potential of these agro-industrial residues for the extraction of cellulose and nanocellulose, which can be further applied as reinforcement in biodegradable films, biocomposites, and sustainable packaging systems [12]. Beyond the inherent abundance and renewability of cellulose in nature, the utilization of agricultural by-products enables the transformation of low-value wastes into high-value materials with significant industrial potential [13]. Moreover, this approach contributes to environmental sustainability by reducing waste accumulation and promoting circular economy practices [5].

Melt extrusion followed by compression molding is particularly attractive as a processing technique due to its industrial scalability and solvent-free nature. Furthermore, that complementary technique plays a decisive role in defining the final microstructure and performance of biocomposites. Nevertheless, processing conditions such as temperature, shear forces, and residence can influence fiber integrity, dispersion, and the crystallinity of the polymer matrix [14]. Therefore, understanding the interaction between processing methods and filler concentration is essential for optimizing composite performance. Melt extrusion and compression molding were selected because they are solvent-free, scalable, and widely used industrial processing techniques for thermoplastic biocomposites. These methods enable efficient dispersion of cellulose microfibers within the PLA/PHBV matrix while preserving the thermoplastic processability of the polymers. Furthermore, their combination allows the production of homogeneous films with controlled thickness and reproducible structural and functional properties.

To the best of the authors’ knowledge, the incorporation of yam, potato, or cassava hull-derived cellulose microfibers as reinforcement of PLA/PHBV biocomposites, for instance, to elucidate how variations in the source, characteristics, and concentration of CM affect interfacial interactions with the polymer matrix and, consequently, the performance of the resulting materials has not been reported yet. Therefore, this study aims to develop PLA/PHBV-based biocomposite films via melt extrusion followed by compression molding and to systematically assess the effect of both cellulose microfiber source (Y, P, and C) and loading level (1 and 3 wt%) on their physicochemical, mechanical, microstructural, optical, thermal, and functional properties to identify a formulation with enhanced performance for sustainable packaging and advanced biodegradable applications.

## 2. Materials and Methods

### 2.1. Materials

PLA (grade LX175; density: 1.25 g·cm^−3^; moisture content < 400 ppm; Mw: 67,000 g·mol^−1^; melting point: 155~170 °C; Tg: 58~62 °C), PHBV powder (density: 1.25 g·cm^−3^; 15 mol% hydroxyvalerate units; Mw: 230,000 g·mol^−1^; melting point: 175~180 °C), and 3-KH-550 silane coupling agent (CAS: 919-30-2; density: 0.94 g·cm^−3^; refractive index: 1.42) were purchased from Zhishang Chemical Co., Ltd. (Jinan, China). Citric acid, sodium hydroxide (NaOH), phosphorus pentoxide (P_2_O_5_), magnesium nitrate, and sodium hypochlorite were supplied by Panreac and Merck (Bogotá, Colombia). Yam, potato, and cassava peels, used as sources of cellulose microfibers, were obtained from a local market in Cartagena. All reagents and solvents were of analytical grade.

### 2.2. Cellulose Microfibers Extraction

The microfiber extraction process from cellulose biomass of agricultural residues (yam, potato, and cassava hulls) follows the methodology proposed by Alemdar & Sain [15] with slight modifications. Firstly, the yam (Y), potato (P) or cassava (C) hulls were cut into 4–5 cm lengths, washed with distilled water and treated with a 0.5% citric acid solution to prevent enzymatic browning. Subsequently, they were dried at 50 °C and ground to facilitate the chemical treatment. After that, an alkaline treatment with 10% NaOH to remove lignin and hemicellulose, followed by a bleaching step using sodium chlorite in an acetate buffer (pH 4.5) was performed, which enabled the obtention of high-purity cellulose. Finally, the purified cellulose was subjected to mechanical homogenization to release the microfibers, which were then stored for subsequent characterization and use. The cellulose microfibers extraction is explained through original images as Supplementary Material for better understanding (Figure S1, see Supplementary Material).

### 2.3. Film Preparation

The formulations were developed according to the specifications outlined in Table 1. Initially, blends of polylactic acid (PLA) and polyhydroxybutyrate (PHBV) at a 75:25 ratio were obtained. Cellulose microfibers (CM) were incorporated into these blends at concentrations of 1 and 3 wt%, along with a silane coupling agent (KH550) to enhance compatibility and dispersion between the PLA/PHBV phases. The biomaterials were processed using the melt-blending technique in a three-section extruder operated at 160, 170, and 180 °C, with a rotor speed of 240 rpm and a residence time of approximately 3 min. After processing, the blends were ground and conditioned for one week at 25 °C and 53% relative humidity (RH) using a magnesium nitrate-6-hydrate oversaturated solution. The films were then fabricated via compression molding using a hot-plate press. For this process, 5 g of the previously conditioned pellets were weighed and placed between Teflon sheets. The molding cycle consisted of preheating at 160 °C for 4 min, a first compression at 50 bar for 1 min, a second compression at 100 bar for 1 min, and a final cooling cycle of 3 min, respectively. The resulting films were re-conditioned at 25 °C and 53% RH until further analysis. Following this period, the characterization analyses were performed [16].

The films were coded as Y-CMn, P-CMn, and C-CMn, where *n* represents the weight percentage of cellulose microfibers (CM) incorporated into the PLA/PHBV matrix, and Y, P, and C correspond to the initial letters of the tuber hull sources (yam, potato, and cassava, respectively). For example, Y-CM3 denotes a PLA/PHBV film reinforced with 3 wt% of CM derived from yam hulls. A neat PLA/PHBV film without CM incorporation was used as the control.

### 2.4. Film Characterization

#### 2.4.1. Thickness

Thickness was measured at five random points across each sample surface using a digital micrometer (TOP EU TL268, Proster Trading Ltd., Hong Kong, China) with an accuracy of 0.001 mm. The average of these measurements was calculated and reported [16].

#### 2.4.2. Moisture Content (MC) and Solubility in Water (Sw)

MC and Sw were determined according to the method adapted from [17]. Film specimens (2 × 2 cm) were initially weighed to establish their wet mass. They were subsequently oven-dried at 60 °C until constant weight to determine the final dry matter. Then, MC was calculated and expressed as a percentage. For solubility in water (Sw) determination, the oven-dried samples were immersed in 25 mL of distilled water at ambient temperature. After a 24 h immersion period, the water was decanted, and the samples were superficially dried with filter paper prior to a final weighing. Sw was then calculated by taking the mass loss of the samples and expressed as grams of solubilized material per 100 g of dry sample [18].

#### 2.4.3. Water Vapor Permeability (WVP)

Water vapor permeability (WVP) was determined following the ASTM E96-9535 gravimetric method [19] with slight modifications. Sample films (diameter = 35 mm) were disposed of on a Payne permeability cup. The relative humidity gradient was 53–100%, which was obtained using a magnesium nitrate oversaturated solution and pure water, respectively. Cups were introduced into desiccators, and these were placed in a temperature-controlled chamber at 25 °C. Mass measurements of the cups were taken at regular intervals using an analytical balance (±0.0001 g). Upon reaching steady-state conditions, the water vapor transmission rate (WVT) was calculated from the slope of the mass loss versus time regression line, normalized by the film’s exposed area [20].

#### 2.4.4. Contact Angle

A drop of distilled water (with dye) was carefully placed at 25 °C onto a film surface (2 × 2 cm) mounted on a horizontal white background. Following a 30 s dwell time, an image was acquired using a digital camera, ensuring the lens was maintained at a fixed distance of 20 cm from the sample. The contact angle was subsequently determined through analysis of the captured image utilizing Gonio trans software version 1.0.3 [21].

#### 2.4.5. Mechanical Properties

The mechanical properties of the films were characterized in accordance with the method described by [22]. The parameters assessed included tensile strength (TS), percentage elongation at break (E), and elastic modulus (EM). Measurements were performed using a texture analyzer (TX-700, Lamy Rheology SARL, Champagne-au-Mont-d’Or, Lyon, France) equipped with a 500 N load cell, at a crosshead speed of 50 mm/min. Specimens, measuring 1 cm in width and 10 cm in length, were tested with an initial grip separation of 5 cm.

#### 2.4.6. Color

The CIELab scale was used to measure the color of the films in a portable colorimeter (CHN Spec CS-10). L*, a*, b* C (Chroma) and h° (hue angle) were measured at random positions over the film surface. The color was then benchmarked against a reference sample to quantify the chromatic variation. This involved calculating the differential values for each coordinate (ΔL*, Δa*, and Δb*). The total color difference (ΔE) was subsequently calculated as shown in Equation (1).

(1) $$\Delta E=\sqrt{{(L}^{*}-{L_{0}^{*})}^{2}+{(a}^{*}-{a_{0}^{*})}^{2}+(b-{b_{0}^{*})}^{2}}$$

where $L_{0}^{*}$, $a_{0}^{*}$, and $b_{0}^{*}$ are values measured for the control film [21].

#### 2.4.7. Gloss

Gloss was measured following the ASTM standard D523 using a flat-surface glossmeter (3NH YG268 multi-angle glossmeter, Minolta, Langenhagen, Germany) at an angle of 60°. Gloss measurements were carried out over a black matte standard plate. A total of three film samples were assessed, with seven replicate measurements taken per sample. The results were expressed as gloss units (GU) [20].

#### 2.4.8. Light Transmission and Opacity

Light transmission of films against ultraviolet and visible light was determined in transmittance mode at selected wavelengths (200–800 nm) using a BIOBASE BK-UV1900 spectrophotometer (Biobase Biodustry Corp., Jinan, China). Film specimens with well-controlled thickness were carefully chosen, cut (1 cm × 3 cm), and attached to the measuring cell for the UV-vis spectrophotometer [21]. The opacity of the films was calculated using Equation (2) and expressed in units of mm^−1^ [16].

(2) $$Opacity=\frac{Absorbance\;(600\;nm)}{Thickness\;(mm)}$$

#### 2.4.9. Thermogravimetric Analysis (TGA)

The thermal stability of the films was evaluated by thermogravimetric analysis (TGA) using a thermogravimetric analyzer (Perkin Elmer Pyris 1, Shelton, CT, USA). Measurements were carried out under a nitrogen atmosphere at a flow rate of 30 mL min^−1^. The mass of the as-prepared samples ranged from 5 to 10 mg. The samples were heated from 25 to 600 °C at a heating rate of 10 °C min^−1^. The weight loss and its derivative (DTG) as a function of temperature were obtained from the TGA curves and subsequently analyzed [23].

#### 2.4.10. Raman Spectroscopy

Raman spectra were acquired using a 532 nm laser excitation with an XploRA Raman microscope (HORIBA Jobin Yvon GmbH, Bensheim, Germany), equipped with a 1200 grooves mm^−1^ grating, providing a spectral resolution of approximately 5 cm^−1^ and a CCD detector with a pixel resolution of about 0.6 cm^−1^. The incident laser power at the sample under a 100× objective was set to 0.1 mW. Raman spectra were measured over 6 scans with an acquisition time of 90 s per scan [24].

#### 2.4.11. Microstructure

The surface microstructure of the films was examined in an optical microscope (ZEISS Model 415500-1800-000, Oberkochen, Germany) at a 10× magnification. For this analysis, samples (1 cm × 1 cm) were prepared to facilitate observation of the surface topography [20].

The cross-sectional analysis was carried out using a scanning electron microscope (JSM-5910, JEOL Ltd., Tokyo, Japan). The film samples were maintained in desiccators with P_2_O_5_ for 2 weeks to guarantee that water was not present in the sample. Film pieces, 0.5 cm^2^ in size, were cryofractured from films, fixed on copper stubs, gold-coated, and observed using an accelerating voltage of 10 kV at 500× magnification [20].

#### 2.4.12. Statistical Analysis

All measures were conducted at least in triplicate, and the data reported in the tables were presented as mean ± standard deviation (mean ± SD). A statistical analysis was carried out using a one-way analysis of variance (ANOVA) approach using the Statgraphics Centurion v.16.1.03 software (StatPoint^®^ Inc., Warrenton, VA, USA). Duncan’s test with a significant 5% level was used to differentiate the statistical significance.

## 3. Results

### 3.1. Physicochemical Properties

#### 3.1.1. Thickness, MC, Sw, WVP, and CAw

The outcomes of thickness, moisture content (MC), water solubility (Sw), water vapor permeability (WVP), and water contact angle (CAw) of the reinforced PLA/PHBV biocomposites are displayed in Table 2. Film thickness differed significantly among formulations (*p* < 0.05), with P-CM1 showing the highest value (248.8 ± 46.08 μm) and Y-CM1 the lowest (172.0 ± 14.73 μm), while the remaining samples were statistically similar to the control.

Thickness variation was mainly associated with the rheological behavior of the blends during processing rather than filler concentration alone. The notable higher thickness of P-CM1 was likely related to the greater aspect ratio and aggregation tendency of potato-derived microfibers, which increased melt viscosity and reduced flowability during compression molding [7]. In contrast, yam-derived CM exhibited better dispersion and more stable rheological behavior, resulting in thinner and more homogeneous films. These findings highlight the importance of controlling filler dispersion and processing conditions to achieve uniform film thickness and ensure reliable interpretation of the reinforcing effects on biocomposite properties [25].

The WVP results revealed that the incorporation of CM significantly (*p* < 0.05) affected the barrier properties of the films, with a strong dependence on the CM source. The control film exhibited the lowest WVP (0.56 g·mm/kPa·h·m^2^), reflecting a dense and homogeneous polymer matrix that restricts water vapor diffusion. In contrast, formulations reinforced with potato-derived CM presented the highest permeability values, regardless of the concentration, suggesting limited interfacial compatibility between those microfibers and the PLA/PHBV matrix. This incompatibility likely promotes the formation of microstructural discontinuities and agglomeration of fibers, creating microchannels that facilitate water transport and, consequently, increase WVP even in thicker films. Additionally, the inherently hydrophilic nature of CM from this source may further enhance water vapor sorption and diffusion through the polymer network [26].

Biocomposites loaded with yam hull-derived CM exhibited intermediate WVP values with no significant differences between concentrations, indicating a possible saturation in microfiber dispersion within the matrix. Notably, C-CM3 showed a significant reduction (*p* < 0.05) in WVP compared to C-CM1, reaching a value close to that of the control film. This improvement may be attributed to better dispersion and interfacial adhesion of cassava hull-derived CM, which promotes a more tortuous diffusion pathway, thereby increasing resistance to vapor transmission and further reducing WVP beyond what thickness alone would predict. These findings suggest that the effectiveness of cellulose microfibers as barrier modifiers is governed primarily by their physicochemical properties, microstructure, and their interfacial interaction and dispersion within the PLA/PHBV matrix, rather than by filler concentration alone [27].

The MC values of the PLA/PHBV films ranged from 8.61% to 11.1% (*p* < 0.05), indicating that the incorporation of CM influenced the water retention behavior of the materials depending on both the source and filler concentration. The control sample exhibited an intermediate MC (9.73%), statistically like most formulations, suggesting that low levels of CM incorporation did not drastically alter the overall moisture balance of the polymer matrix. Y-CM1 and C-CM1 presented the lowest MC (8.69% and 8.61%, respectively), indicating that the addition of 1 wt% CM may have promoted a more compact structure with reduced water uptake. This could be associated with better dispersion of the microfibers at low concentrations, improving interfacial interactions between the CM and PLA/PHBV matrix and limiting free volume available for moisture absorption [28]. However, at higher filler loadings, the greater availability of hydrophilic sites and possible filler agglomeration may facilitate moisture retention within the matrix [28]. This effect was particularly evident in the C-CM3 sample, which exhibited the highest MC value (11.1%). These findings indicate that increasing CM concentration from 1 to 3 wt% generally increased moisture content, particularly for cassava-derived CM, highlighting the importance of composition, interfacial adhesion, dispersion, and the intrinsic hydrophilicity of CM source in determining the hygroscopic behavior of the PLA/PHBV biocomposites [3].

The Sw behavior was predominantly influenced by the CM concentration, regardless of its source. Y-CM3 and P-CM3 samples exhibited significantly lower solubility values (*p* < 0.05), suggesting that higher microfiber loadings enhance the structural integrity of the films and reduce the leaching of soluble components. This behavior can be associated with the high density of hydroxyl groups in cellulose, which promotes intermolecular interactions, both within the filler and with the polymer matrix, leading to the formation of a more cohesive and compact network that limits water penetration and dissolution, since even though MC is hydrophilic, when well embedded, it does not easily dissolve out [29]. In contrast, the control and Y-CM1, P-CM1, and C-CM1 showed the highest solubilities, with no significant differences among them (*p* > 0.05), indicating a more open matrix structure that facilitates the dissolution of water-soluble components. Like the trends for WVP, these results indicate that Sw is governed not only by microfiber concentration but also by their dispersion and interfacial compatibility with the PLA/PHBV matrix [30].

The CAw measurements revealed that the surface wettability of the biocomposites was primarily influenced by the CM source. The control and C-CM formulations exhibited the lowest (*p* < 0.05) contact angle values (~66°), reflecting a relatively hydrophilic surface. In contrast, Y-CM and P-CM samples presented a noticeable increase in CAw, reaching ~78° for Y-CM3 film, which suggests enhanced surface hydrophobicity. These differences can be attributed to the interactions between yam- or potato-derived cellulose microfibers and the PLA/PHBV matrix, which can modify both surface energy and microstructural topography. Specifically, the incorporation of these last microfibers may generate a more textured surface with micro-scale irregularities capable of trapping air pockets, thereby increasing the apparent contact angle and promoting hydrophobic behavior [31]. Furthermore, the higher CAw observed for biocomposites reinforced with Y-CM3 correlates with its lower Sw, indicating a more compact structure and greater resistance to initial water wetting. This behavior is likely associated with improved dispersion and stronger interfacial interactions, which favor the reorganization of less polar components at the film surface and reduce the exposure of hydrophilic groups [27].

#### 3.1.2. Mechanical Properties

The results of Table 3 showed a strong concentration-dependent trend, mainly for E and EM. Increasing microfiber concentration from 1 to 3 wt% has shown limited or moderate effects on TS. At low CM (1%) loading, it maintained or slightly enhanced strength (35–38 MPa) without severe deterioration, as compared to the control film, and this suggests good stress transfer between the microfibers and biopolymers, as well as an acceptable dispersion of CM within the matrix. Conversely, high CM (3%) loading tended to reduce TS. According to Marmol et al. [8], the addition of fibers to non-polar thermoplastics reduces the tensile strength owing to the poor fiber–matrix adhesion at the interface, which results in low interfacial stress transfer. Then, to enhance the reinforcing effect of the fibers within these polymers, the use of coupling agents becomes crucial. This behavior is typical for CM-reinforced composites where rigid fillers naturally enhance stiffness but often reduce ductility [8].

As expected, at high CM loading (3 wt%), the elongation decreased sharply demonstrating increased brittleness of the biocomposites. It indicates that higher microfiber content could promote CM aggregation, restrict polymer chain mobility, lower flexibility, or reduce the ability to deform plastically before fracture [3]. Among all formulations, P-CM3 exhibited the most brittle behavior and the lowest elongation at break (3.5%), representing a reduction of approximately 44% compared to the control.

The incorporation of cellulose microfibers significantly enhanced the EM of PLA/PHBV films, indicating effective reinforcement of the polymer matrix. All reinforced films showed a higher EM than the control, and this property increased consistently with higher CM loading. The biocomposites reinforced with cassava CM exhibited the most favorable mechanical performance, particularly C-CM3, which showed the highest (*p* < 0.05) stiffness (2050 MPa), relatively high TS and moderate elongation compared with other samples, indicating stronger matrix–fiber interaction and better fiber dispersion [8]. Overall, high cellulose microfiber content increased the elastic modulus but reduced tensile strength and elongation at break, likely due to the poor fiber dispersion and adhesion as can be observed in the morphological micrographs in Section 3.1.5 [3].

#### 3.1.3. Gloss and Optical Properties

Results from Table 4 demonstrate a clear and statistically significant effect (*p* < 0.05) of both CM origin (Y, P, and C) and loading level (1 and 3 wt%) on the gloss and optical properties of the films.

As expected, the control exhibited the highest gloss value (62 GU), indicative of a smooth and highly reflective surface characteristic of neat PLA/PHBV matrices. The incorporation of CM consistently (*p* < 0.05) reduced gloss compared to the control, confirming that the presence of fillers disrupts surface uniformity and increases light scattering. A pronounced concentration-dependent effect was observed, as increasing the CM content from 1 to 3 wt% led to a significant decrease in gloss for all microfiber sources. This trend was particularly evident in formulations containing yam- and potato-derived CM, where gloss values decreased sharply from 56.3 (Y-CM1) to 18.7 (Y-CM3) and from 38.0 (P-CM1) to 20.7 (P-CM3), respectively. These results suggest that higher filler loading could promote increased surface roughness, heterogeneity, and possibly CM agglomeration, which enhances diffuse reflection and reduces specular gloss [32].

Regarding the color parameters, the Control, P-CM3, C-CM1, and C-CM3 samples exhibited high L* values (~86) with no significant differences (*p* > 0.05), indicating high brightness and minimal light scattering with negligible color alteration. These results suggest that the incorporation of potato and cassava-hull-derived CM, even at higher loadings, does not substantially affect the luminosity of the PLA/PHBV biocomposites. Conversely, the incorporation of yam hull-derived CM significantly reduced the lightness of the films while intensifying their chromatic characteristics, particularly at higher concentrations. Specifically, Y-CM3 displayed increased a* (redness) and b* (yellowness) values, along with the highest chroma (C = 12.2) and a pronounced total color difference (ΔE = 23), indicating a clearly perceptible color change. Additionally, the hue angle (h ≈ 90°) reflects a more marked shift toward reddish–yellow tones [33].

Like Y-CM reinforced films, P-CM3 and C-CM3 samples exhibited a shift toward reddish (positive a*) and yellowish (positive b*) tones, indicating that cellulose microfibers from all sources contain chromophore compounds. These may include residual lignin as well as naturally occurring pigments such as flavonoids and carotenoids, whose influence becomes more pronounced at higher filler loadings. When considered alongside the L*, a*, and chroma results, it is evident that the optical behavior of the films is governed by a combination of factors, including fiber morphology, particle size, chemical composition, and dispersion within the PLA/PHBV matrix. Among these parameters, the b* coordinate provides the most direct indication of pigment-related contributions to color development [33].

Regarding hue angle (h°), the control film (282.9°) falls within the blue–green region and serves as a baseline for comparison. A wide variation in hue (90° to 323°) was observed among the formulations, confirming that both CM source and concentration significantly influence film tonality. Particularly, Y-CM3 and P-CM1 samples exhibited a pronounced shift toward the yellow–red region (90–105.9°), whereas P-CM3 and C-CM3 shifted toward the blue–violet region (~323°). The remaining formulations showed hue values comparable to the control, indicating minimal chromatic alteration. These variations in h° values can be attributed to differences in CM composition, particle size, and dispersion, as well as interfacial interactions within the PLA/PHBV matrix. Additionally, processing conditions may contribute to these changes by affecting light scattering and reflectance, modifying the optical path and refractive index, or inducing chemical transformations such as oxidation or Maillard-type reactions, particularly at higher CM loadings [34].

Finally, in terms of total color difference (ΔE), films reinforced with yam-derived CM exhibited pronounced deviations from the control (ΔE > 10), indicating substantial alterations in optical properties, whereas those reinforced with cassava-derived CM displayed minimal color differences (ΔE ≈ 1–2), suggesting that these formulations remain visually similar to the reference sample and retain greater stability in appearance. Overall, all these results demonstrate that the CM source plays a decisive role in determining the final color of the PLA/PHBV biocomposites. Particularly, microfibers derived from yam and potato tend to induce more yellowish tones and higher ΔE values. The incorporation of Y-CM3 and P-CM1, for instance, enhances the diffusion of incident light, leading to reduced gloss and lightness, along with increased chromatic intensity. This behavior may be attributed to the presence of residual compounds, e.g., lignin or pigments, and differences in cellulose morphology and crystallinity [34].

Opacity results reflect the extent of light attenuation within films and, therefore, provide direct insight into their transparency. As expected, the control film exhibited the lowest opacity (0.3 mm^−1^, *p* < 0.05), confirming a highly transparent matrix with minimal light scattering. In contrast, opacity increased with CM incorporation, with concentration exerting a dominant effect regardless of CM source. Biocomposites reinforced with cassava-derived CM showed the highest opacity values (~1.7 mm^−1^) at both loadings, with no significant differences among concentrations (*p* > 0.05). The observed decrease in transparency is likely associated with increased light scattering arising from microfiber loading due to refractive index mismatch between the dispersed phase and the PLA/PHBV matrix, as well as possible filler agglomeration at higher loadings. Conversely, lower opacity at reduced CM content suggests improved dispersion and smaller effective particle size within the matrix [35]. These results align well with the gloss data, reinforcing that optical performance is governed by surface morphology and internal light scattering, both strongly dependent on filler content, specific source and dispersion state within the PLA/PHBV matrix.

#### 3.1.4. Transmittance

**Figure 1:**
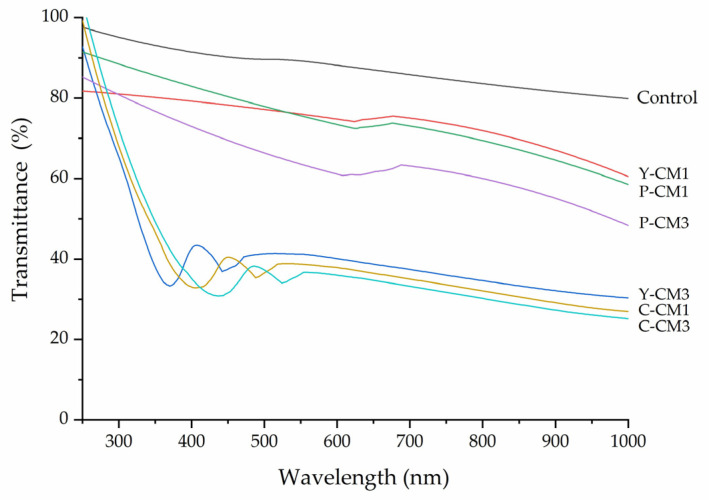
Direct UV-vis transmittance spectra of the different treatments.

Figure 1 presents the UV-vis direct transmittance spectra of the PLA/PHBV biocomposites. The control exhibited the highest (*p* < 0.05) light transmittance values (80–95%) across the entire spectral range, confirming its high optical transparency. In contrast, films incorporated with CM derived from yam, potato, or cassava showed a progressive decrease in transmittance, which can be attributed to increased light scattering caused by the presence of dispersed microfibers, differences in particle size or the formation of microstructural heterogeneities within the polymer matrix [36]. Y-CM3, C-CM1, and C-CM3 drastically reduced transmittance (*p* < 0.05) in both the ultraviolet (UV < 350 nm) and visible (350–800 nm) regions. The pronounced attenuation observed in these spectra is likely associated with the presence of chromophore groups, such as lignin residues and carbonyl functionalities, which enhance light absorption. Additionally, possible structural rearrangements or interactions during processing may promote light scattering. As a result, those samples exhibited the highest absorption and lowest transmittance in the UV and visible regions, respectively, demonstrating improved barrier properties against light transmission compared to the control. According to Caydamli et al. [36], microfiber loading tends to increase opacity because the refractive index mismatch between the fibers and polymer matrix promotes scattering of incident light.

Although the optical response of the reinforced films followed a similar trend without drastic differences among the curves, the slight reductions in transmittance confirm that besides the refractive index mismatch, there are multiple other influencing factors on the transmission of PLA/PHBV biocomposites, such as surface roughness, thickness, fiber sizing, interfacial compatibility, defects, and voids [36].

#### 3.1.5. Microstructure

**Figure 2:**
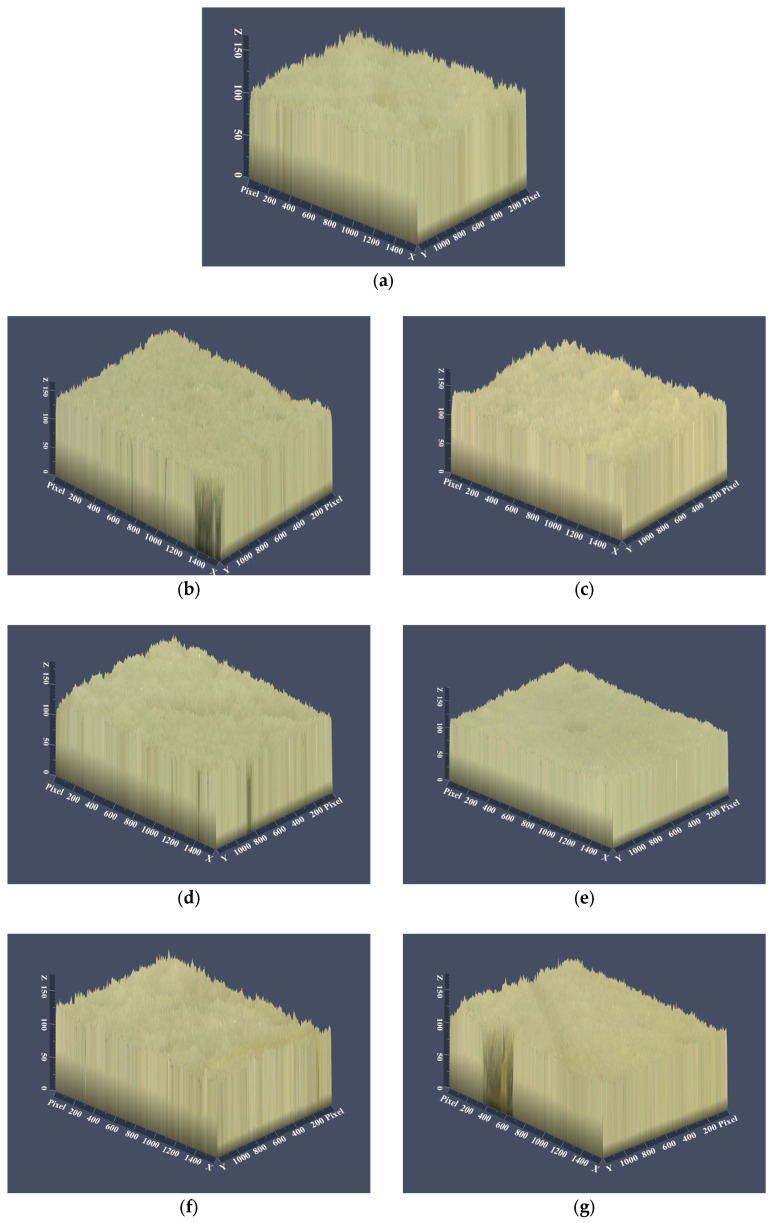
2.5D surface optical micrographs (10×) of (**a**) control film and cellulose microfiber-reinforced PLA/PHBV biocomposites; (**b**,**c**) Y-CM; (**d**,**e**) P-CM; and (**f**,**g**) C-CM at 1 and 3 wt%, respectively.

The 2.5D optical surface micrographs (Figure 2) reveal the topographical features of the films, where height variations (Z-axis) reflect surface roughness. As expected, the control (Figure 2a) exhibited a smooth, continuous, and homogeneous morphology with no pronounced defects such as cracks or voids, indicating a well-formed PLA/PHBV matrix. The incorporation of CM induced noticeable morphological changes (Figure 2b–g). Films loaded with yam-derived CM displayed heterogeneous surfaces characterized by combined macro-scale waviness and micro-scale roughness, mainly in the Y-CM3 sample, where higher filler loading promoted microfiber agglomeration and non-uniform dispersion. These features are consistent with the observed reductions in gloss, lightness, and transparency, as well as increased contact angle values. Conversely, formulations reinforced with potato-derived CM presented a comparatively smoother and more homogeneous surface, suggesting improved dispersion and stable matrix–filler interactions. Finally, films loaded with cassava-derived CM displayed a more pronounced, jagged surface due to microfibers partially protruding from the matrix. However, the relatively uniform distribution of these features indicates effective dispersion without large agglomerates. This more organized morphology was associated with stable optical properties, good structural integrity, and barrier performance comparable to the control [37].

**Figure 3:**
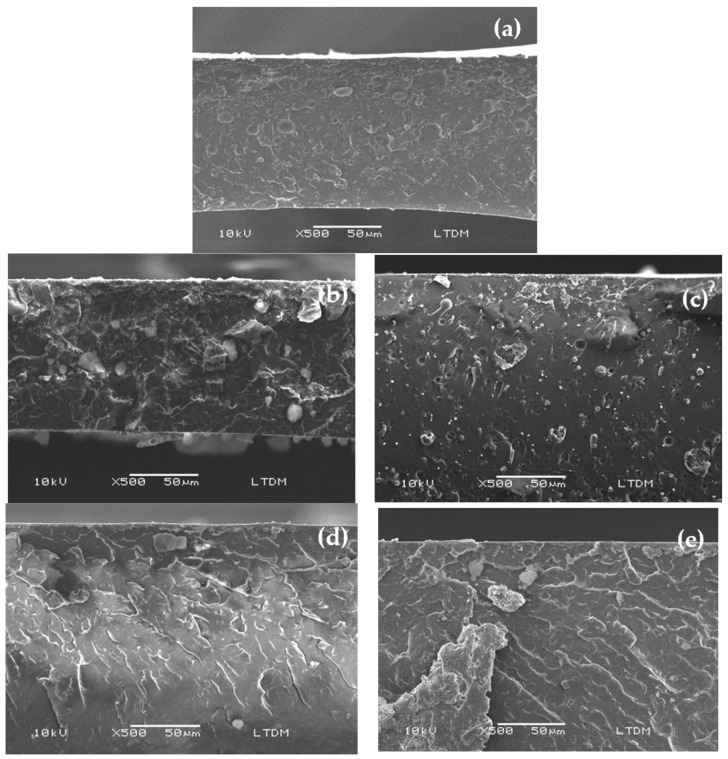

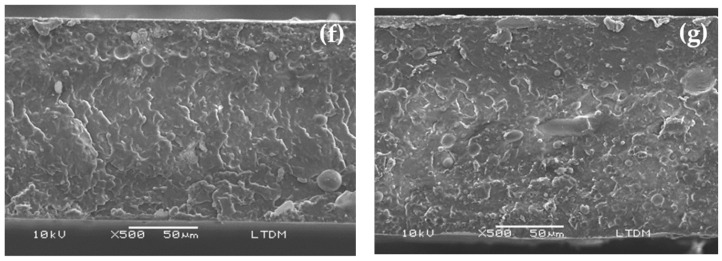
SEM micrographs of the cross-sections of (**a**) control film and cellulose microfiber-reinforced PLA/PHBV biocomposites; (**b**,**c**) Y-CM; (**d**,**e**) P-CM; and (**f**,**g**) C-CM at 1 and 3 wt%, respectively.

SEM micrographs of the cross-sections revealed that the control PLA/PHBV film possessed a smooth and compact morphology with a relatively homogeneous matrix structure (Figure 3a). The fracture surface appears continuous and dense, indicating good miscibility between the PLA and PHBV phases. Only a limited number of small voids and spherical cavities are observed, suggesting minimal internal defects and good structural integrity. This morphology aligns well with the mechanical properties discussed in Section 3.1.2, since smooth compact structures generally permit greater polymer chain mobility and fewer rigid inclusions leading to better flexibility [3].

Incorporation of 1 wt% cellulose microfiber caused a rougher fracture surface with well-dispersed fibrillar structures, suggesting improved interfacial interactions and stress transfer that achieved a more optimal reinforcement state for Y-CM1, P-CM1, and C-CM1, whereas excessive loading (3 wt%) appears to promote CM agglomeration, greater void density, and interfacial discontinuities, negatively affecting the structural homogeneity for Y-CM3, P-CM3, and C-CM3, indicating that excessive CM loading may disrupt matrix continuity, promote brittle fracture behavior, and less compact structure, which is typical in natural fiber-reinforced biocomposites where excessive filler loading compromises dispersion quality. Although the matrix remains relatively continuous as seen in Figure 3c,g, the increased porosity suggests deterioration in filler dispersion and weaker interfacial bonding compared to 1 wt% loading films (Figure 3b,d,f). Particularly, P-CM3 (Figure 3e) displays a markedly different morphology with a large irregular protrusion, an increased surface roughness, and evidence of microfiber clustering. These defects reduced tensile strength and elongation due to easier crack initiation and propagation. Therefore, the addition of greater CM content modifies the internal morphology of the PLA/PHBV blend by increasing roughness and inducing dispersed domains within the matrix [7].

#### 3.1.6. Thermogravimetric Analysis (TGA)

TGA provides insight into the thermal stability and degradation behavior of PLA/PHBV biocomposite, distinguishing early thermal events (Td_1_), degradation onset (Td_2_), and the main degradation stage (Td_3_) (Table 5). All samples exhibited a typical multi-step degradation profile characteristic of PLA/PHBV systems [4]. The initial mass loss (Td_1_ ≈ 63–82 °C) is attributed to moisture evaporation and plasticization effects, which are more evident at higher CM content due to their hydrophilic nature. The onset of major degradation (Tonset ≈ 280–305 °C), closely matching Td_2_ (≈287–303 °C), corresponds to the decomposition of the polymer matrix and less stable lignocellulosic fractions. The main degradation stage (Tmax ≈ 340–370 °C; Td_3_ ≈ 368–377 °C) is associated with PLA/PHBV chain scission. The relative stability of Td_3_ across formulations indicates that the degradation mechanism is primarily governed by the polymer backbone, with CM acting mainly as a physical modifier [4,23]. Minor variations in Tonset and Tmax suggest that low CM loadings may slightly enhance thermal stability; they function as nucleating agents that promote crystallization and improve interfacial interactions within the polymer matrix [3], while higher contents can induce earlier degradation due to agglomeration and interfacial defects. The increased residual mass in reinforced biocomposites further confirms their contribution to char formation, which may act as a protective barrier during thermal decomposition. Overall, the CM source does not alter the fundamental degradation pathway of PLA/PHBV, but CM incorporation modulates its thermal response depending on filler content and dispersion [23].

**Figure 4:**
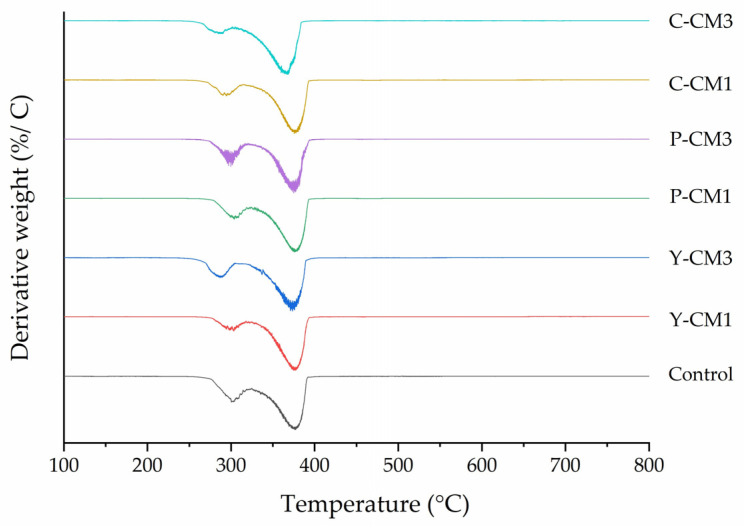
Derivative thermogravimetric curves (%/°C) as function of temperature (°C) of cellulose microfiber-reinforced PLA/PHBV biocomposite films.

Figure 4 presents the derivative thermogravimetric (DTG) curves of the control and CM-reinforced PLA/PHBV biocomposites as a function of temperature. All samples exhibited a two-step degradation pattern, confirming the multi-component nature of the systems. The first degradation peak, observed in the range of 280–320 °C, is attributed to the decomposition of hemicellulose and amorphous cellulose fractions, as well as the initial degradation of less thermally stable PHBV domains. The presence of cellulose microfibers appears to influence the degradation kinetics, likely by modifying polymer chain mobility and interfacial interactions within the matrix, which in turn affects the rate and progression of thermal decomposition [23]. The second and most prominent DTG peaks, located between 330 and 400 °C, remained nearly unchanged across all formulations. These peaks are primarily associated with the degradation of the PLA matrix, the decomposition of the crystalline PHBV phase, and the depolymerization of cellulose. This observation is particularly relevant, as it indicates that although CM incorporation may shift the onset of degradation (Tonset) to lower temperatures, it does not significantly affect the main degradation stage of the PLA/PHBV matrix. Notably, samples exhibiting slightly higher Tmax values, i.e., the C-CM1 sample, suggest improved thermal stability, likely due to enhanced interfacial interactions and potential nucleating effects. Conversely, formulations with lower Tmax, i.e., P-CM3, may experience reduced thermal stability because of microfiber agglomeration and interfacial defects [38].

#### 3.1.7. Raman Spectroscopy

**Figure 5:**
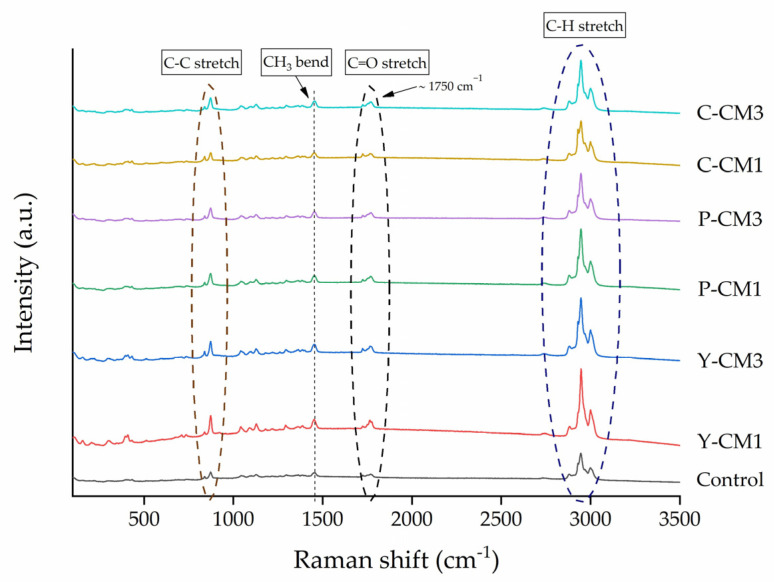
Raman spectra of cellulose microfiber-reinforced PLA/PHBV biocomposite films.

The Raman spectra (Figure 5) reveal clear differences among PLA/PHBV biocomposites as a function of CM source and loading, reflecting changes in molecular organization, crystallinity, and interfacial interactions. Characteristic bands of the PLA/PHBV matrix were consistently observed in all samples, including signals at 840–900 cm^−1^ (C–C stretching), 1040–1120 cm^−1^ (C–O–C bond vibrations), ~1450 cm^−1^ (CH_3_ bending), ~1750 cm^−1^ (C=O stretching), and 2800–3000 cm^−1^ (C–H stretching). The preservation of these bands confirms that the chemical structure of the polymer matrix remains unaffected by CM incorporation and processing [39]. Nonetheless, variations in peak intensity and bandwidth, particularly in the 1000–1200 cm^−1^ region, indicate contributions from cellulose (C–O and C–C vibrations) and suggest differences in dispersion and interfacial interactions. Increased intensity and slight broadening of the carbonyl band (~1750 cm^−1^) in films reinforced with CM, regardless of the source, point to interactions between cellulose hydroxyl groups and the ester functionalities of the PLA/PHBV matrix [40].

Differences among CM sources are evident in spectral definition and baseline behavior. For instance, broader and less defined peaks in C-CM1 suggest poorer dispersion and possible microfiber agglomeration, leading to structural heterogeneity. In contrast, sharper and more defined peaks observed in films reinforced with yam- and potato-derived CM may indicate improved dispersion and stronger interfacial compatibility. Furthermore, subtle changes in carbonyl band intensity and peak broadening in Y-CM1, Y-CM3, and P-CM1 samples may also reflect reduced crystallinity due to restricted chain mobility, whereas more defined features could indicate nucleating effects that promote ordered regions within the matrix. Overall, Raman analysis confirms that while CM incorporation does not alter the chemical identity of PLA/PHBV films, it significantly influences molecular organization, phase interactions, and structural homogeneity, with these effects strongly dependent on microfiber source and dispersion quality [40].

## 4. Conclusions

The incorporation of CM significantly modified the structural, thermal, mechanical, barrier, and optical properties of the films; however, the extent of these effects strongly depended on both the microfiber source and loading level. The addition of CM promoted polymer crystallization, modified spherulitic morphology, and improved the compactness and interfacial interactions within the matrix. Low CM incorporation (1 wt%) generally produced the most favorable balance of properties, while excessive loading (3 wt%) increased rigidity but negatively affected structural homogeneity and mechanical performance. In particular, the films showed thicknesses ranging from approximately 172 to 248 µm, and moisture content remained relatively stable among formulations (8.61–10.10%). Optical characterization revealed that all films maintained relatively similar transmittance profiles, and mechanical analysis confirmed the reinforcing effect of cellulose microfibers on the PLA/PHBV matrix.

Among all formulations, films reinforced with CM derived from cassava hulls exhibited the most balanced overall performance, combining enhanced barrier behavior, good microfiber dispersion, improved structural compactness, improved stiffness without substantial loss of ductility, and minimal optical alteration. These characteristics make cassava-derived CM particularly promising for sustainable and eco-friendly packaging applications. The results demonstrate that controlled CM incorporation is an effective strategy to tailor the properties of neat biodegradable PLA/PHBV biocomposites. Nonetheless, future studies should focus on long-term biodegradation under realistic disposal conditions, scale-up processability, and surface modification strategies to further improve toughness, mechanical resistance, and moisture barrier properties while preserving compostability and recyclability of bio-based composites.

## Figures and Tables

**Table 1:** Mass fractions of CM-reinforced PLA/PHBV biocomposite formulations.

Formula	PLA	PHBV	CM (Y)	CM (P)	MC (C)	KH550
Control	0.7478	0.2493	0.0000	0.0000	0.0000	0.0030
Y-CM1	0.7404	0.2468	0.0099	0.0000	0.0000	0.0030
Y-CM3	0.7260	0.2420	0.0290	0.0000	0.0000	0.0029
P-CM1	0.7404	0.2468	0.0000	0.0099	0.0000	0.0030
P-CM3	0.7260	0.2420	0.0000	0.0299	0.0000	0.0029
C-CM1	0.7404	0.2468	0.0000	0.0000	0.0099	0.0030
C-CM3	0.7260	0.2420	0.0000	0.0000	0.0290	0.0029

**Table 2:** Physicochemical properties of cellulose microfiber-reinforced PLA/PHBV biocomposite films.

Sample	Thickness (μm)	WVP (g·mm/kPa·h·m^2^)	MC (%)	Sw (%)	CAw (°)
Control	189.3 ± 5.51 ^ab^	0.56 ± 0.01 ^e^	9.73 ± 0.33 ^ab^	3.41 ± 0.56 ^a^	64.3 ± 2.36 ^d^
Y-CM1	172.0 ± 14.73 ^b^	0.76 ± 0.01 ^c^	8.69 ± 0.90 ^b^	3.32 ± 0.70 ^a^	74.3 ± 1.15 ^b^
Y-CM3	192.0 ± 11.79 ^ab^	0.76 ± 0.05 ^c^	10.1 ± 0.67 ^ab^	1.65 ± 0.32 ^b^	77.7 ± 0.58 ^a^
P-CM1	248.8 ± 46.08 ^a^	1.19 ± 0.03 ^a^	9.53 ± 0.59 ^b^	3.00 ± 0.57 ^ab^	71.7 ± 1.53 ^bc^
P-CM3	232.7 ± 29.14 ^ab^	1.14 ± 0.06 ^a^	9.99 ± 0.80 ^ab^	1.64 ± 1.05 ^b^	70.7 ± 0.58 ^c^
C-CM1	216.3 ± 37.61 ^ab^	0.87 ± 0.01 ^b^	8.61 ± 1.16 ^b^	2.08 ± 0.71 ^ab^	66.7 ± 1.53 ^d^
C-CM3	225.0 ± 47.03 ^ab^	0.64 ± 0.02 ^d^	11.1 ± 0.69 ^a^	2.21 ± 0.96 ^ab^	66.3 ± 1.53 ^d^

Mean values ± standard deviation. *n* = 3. Different letters in the same column indicate significant differences (*p* < 0.05). WVP: water vapor permeability. MC: moisture content. Sw: solubility in water. CAw: contact angle.

**Table 3:** Mean values and standard deviation of mechanical properties (TS: tensile strength. E: elongation at break. EM: elastic modulus) of cellulose microfiber-reinforced PLA/PHBV biocomposite films.

Sample	TS (MPa)	E (%)	EM (MPa)
Control	34 ± 3 ^ab^	6.2 ± 0.5 ^a^	1550 ± 85 ^d^
Y-CM1	37.0 ± 2 ^a^	5.6 ± 0.4 ^ab^	1720 ± 78 ^c^
Y-CM3	33 ± 4 ^ab^	3.8 ± 0.3 ^cd^	1950 ± 24 ^ab^
P-CM1	35 ± 3 ^ab^	5.2 ± 0.2 ^b^	1680 ± 120 ^cd^
P-CM3	31.5 ± 2 ^b^	3.5 ± 0.2 ^d^	1900 ± 64 ^b^
C-CM1	38 ± 3 ^a^	5.4 ± 0.4 ^ab^	1780 ± 71 ^bc^
C-CM3	36.5 ± 3 ^a^	4.1 ± 0.3 ^c^	2050 ± 80 ^a^

Mean values ± standard deviation. *n* = 3. Different letters in the same column indicate significant differences (*p* < 0.05). TS: tensile strength. E: elongation at break. EM: elastic modulus.

**Table 4:** Gloss and optical properties of reinforced PLA/PHBV biocomposite films.

Sample	Gloss (GU)	L*	a*	b*	C	h°	ΔE	Opacity (mm^−1^)
Control	62.0 ± 1.7 ^a^	86.0 ± 0.26 ^a^	0.19 ± 0.44 ^ab^	−1.03 ± 0.36 ^d^	1.05 ± 0.35 ^c^	282.9 ± 1.58 ^b^	---	0.30 ± 0.01 ^d^
Y-CM1	56.3 ± 1.2 ^b^	82.83 ± 1.37 ^b^	0.36 ± 0.28 ^ab^	12.2 ± 0.83 ^a^	0.56 ± 0.26 ^c^	210.8 ± 0.26 ^c^	14.20 ± 1.1 ^c^	0.49 ± 0.17 ^c^
Y-CM3	18.7 ± 1.2 ^e^	67.44 ± 0.44 ^c^	0.47 ± 0.25 ^a^	12.6 ± 0.46 ^a^	12.2 ± 0.82 ^a^	90.05 ± 1.13 ^d^	23.00 ± 0.61 ^a^	1.13 ± 0.00 ^b^
P-CM1	38.0 ± 0.0 ^d^	68.96 ± 1.26 ^c^	−2.81 ± 0.80 ^c^	6.94 ± 0.88 ^b^	7.56 ± 0.53 ^b^	105.89 ± 0.46 ^d^	18.97 ± 0.96 ^b^	0.53 ± 0.12 ^c^
P-CM3	20.7 ± 1.5 ^e^	85.96 ± 0.24 ^a^	0.59 ± 0.14 ^a^	0.46 ± 0.19 ^c^	0.77 ± 0.19 ^c^	323.18 ± 3.11 ^a^	1.18 ± 0.20 ^d^	0.98 ± 0.15 ^b^
C-CM1	47.0 ± 6.9 ^c^	86.54 ± 0.47 ^a^	−0.39 ± 0.62 ^b^	−1.12 ± 0.31 ^d^	1.02 ± 0.46 ^c^	247.9 ± 0.24 ^bc^	1.28 ± 0.29 ^d^	1.64 ± 0.01 ^a^
C-CM3	44.3 ± 3.2 ^c^	85.37 ± 0.25 ^a^	0.64 ± 0.11 ^a^	0.28 ± 0.10 ^c^	0.96 ± 0.27 ^c^	323.57 ± 0.27 ^a^	1.86 ± 0.24 ^d^	1.72 ± 0.01 ^a^

Mean values ± standard deviation. *n* = 3. Different letters in the same column indicate significant differences (*p* < 0.05).

**Table 5:** Thermal parameters of cellulose microfiber-reinforced PLA/PHBV biocomposite films.

Sample	Td_1_ (T_initial_)	Td_2_ (T_onset_)	Td_3_ (T_max_)
Control	65.55 ± 0.2 ^c^	300.4 ± 0.3 ^a^	377.25 ± 0.2 ^a^
Y-CM1	82.13 ± 0.4 ^a^	303.0 ± 0.1 ^a^	376.97 ± 0.1 ^a^
Y-CM3	66.36 ± 0.2 ^c^	287.1 ± 0.3 ^ab^	373.44 ± 0.3 ^a^
P-CM1	73.93 ± 0.3 ^b^	303.1 ± 0.2 ^a^	375.65 ± 0.3 ^a^
P-CM3	66.04 ± 0.3 ^c^	299.7 ± 0.2 ^a^	374.83 ± 0.3 ^a^
C-CM1	68.98 ± 0.2 ^b^	294.7 ± 0.2 ^ab^	375.52 ± 0.2 ^a^
C-CM3	63.13 ± 0.2 ^d^	286.9 ± 0.2 ^ab^	368.08 ± 0.1 ^b^

Mean values ± standard deviation. *n* = 3. Different letters in the same column indicate significant differences (*p* < 0.05).

## Data Availability

The original contributions presented in this study are included in the article; further inquiries can be directed to the corresponding author.

## Supplementary Materials

The following supporting information can be downloaded at: https://www.mdpi.com/article/10.3390/polym18111350/s1, Figure S1: Schematic representation of the cellulose microfiber extraction from cassava hulls.
