# Supplementary material for: Development of Poly(lactic acid)/Poly(3-hydroxybutyrate-co-3-hydroxyvalerate) Biocomposite Films: Influence of Cellulose Microfiber Source on Structural and Functional Properties
Source: Polymers (Basel). 2026 May 29;18(11):1350. doi: 10.3390/polym18111350 (PMC13258846; doi:10.3390/polym18111350)

Figure S1. Schematic representation of the cellulose microfiber extraction from cassava hulls

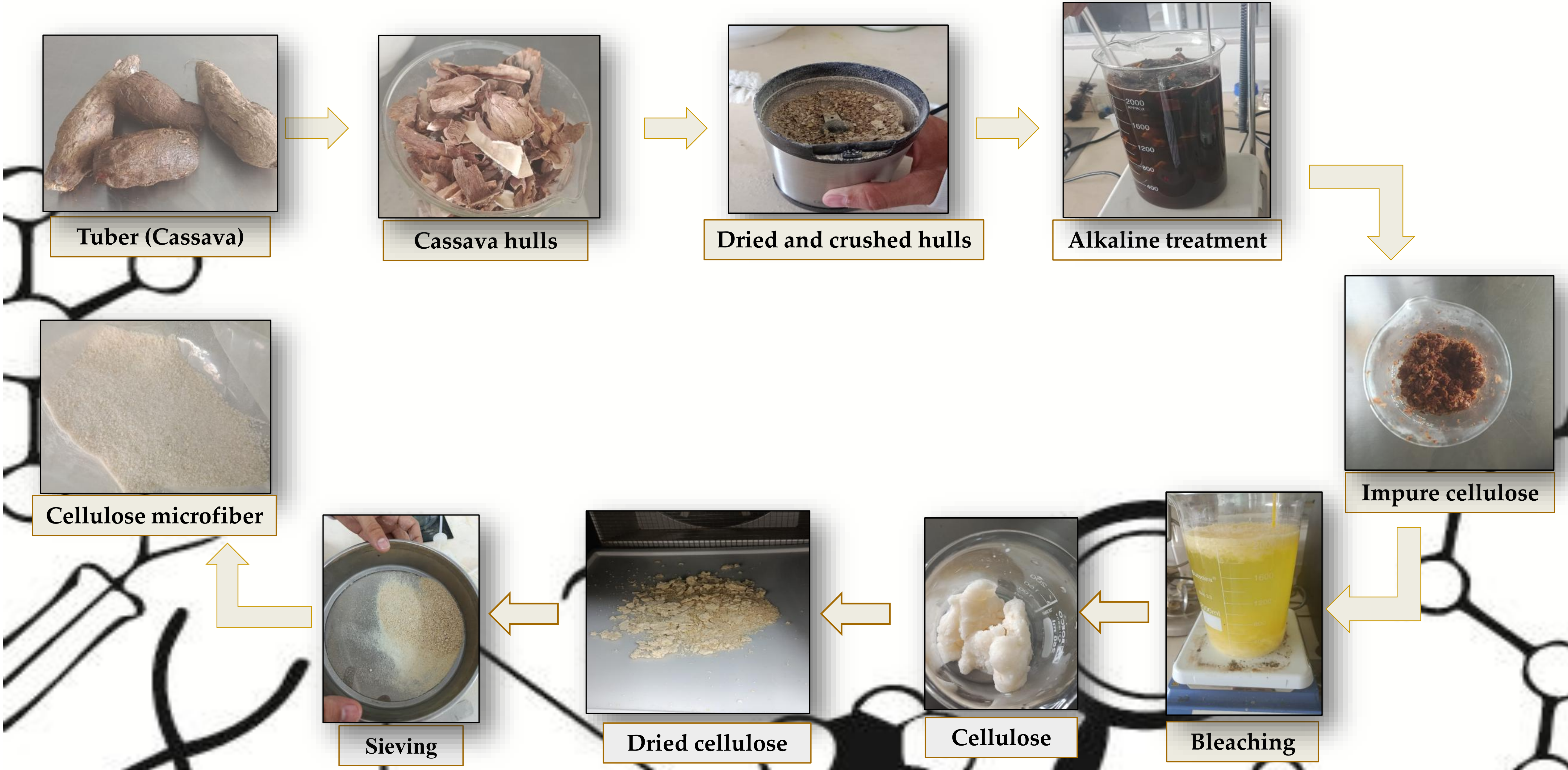

Supplement: Supplementary file 1 [file polymers-18-01350-s001.zip › polymers-4287582-supplementary.pdf]
